# Variables Influencing the Accuracy of 3D Modeling of Existing Roads Using Consumer Cameras in Aerial Photogrammetry

**DOI:** 10.3390/s18113880

**Published:** 2018-11-11

**Authors:** Juan J. González-Quiñones, Juan F. Reinoso-Gordo, Carlos A. León-Robles, José L. García-Balboa, Francisco J. Ariza-López

**Affiliations:** 1 TargetPix, 18015 Granada, Spain; juanjogonqui@gmail.com; 2 Department Architectural and Engineering Graphic Expression, University of Granada, 18071 Granada, Spain; cleon@ugr.es; 3 Department Cartographic, Geodesic and Photogrammetric Engineering, University of Jaén, 23071 Jaén, Spain; jlbalboa@ujaen.es (J.L.G.-B.); fjariza@ujaen.es (F.J.A.-L.)

**Keywords:** photogrammetry, camera, accuracy model, modulation transfer function, neural network

## Abstract

Point cloud (PC) generation from photogrammetry–remotely piloted aircraft systems (RPAS) at high spatial and temporal resolution and accuracy is of increasing importance for many applications. For several years, photogrammetry–RPAS has been used to recover civil engineering works such as digital elevation models (DEMs), triangle irregular networks (TINs), contour levels, orthophotographs, etc. This study analyzes the influence of variables involved in the accuracy of PC generation over asphalt shapes and determines the most influential variable based on the development of an artificial neural network (ANN) with patterns identified in the test flights. The input variables were those involved, and output was the three-dimension root mean square error (3D-RMSE) of the PC in each ground control point (GCP). The result of the study shows that the most influential variable over PC accuracy is the modulation transfer function 50 (MTF50). In addition, the study obtained an average 3D-RMSE of 1 cm. The results can be used by the scientific and civil engineering communities to consider MTF50 variables in obtaining images from RPAS cameras and to predict the accuracy of a PC over asphalt based on the ANN developed. Also, this ANN could be the beginning of a large database containing patterns from several cameras and lenses in the world market.

## 1. Introduction

Point cloud generation from photogrammetry– remotely piloted aircraft systems (RPAS) at high spatial and temporal resolution and accuracy is of increasing importance for many applications [1,2]. The improvement is such that photogrammetry–RPAS is a robust tool to retrieve topographic products such as digital elevation models (DEMs), triangle irregular networks (TINs), contour levels, and orthophotographs [3,4,5,6,7,8]. Also, RPAS spends less time on photogrammetric data acquisition, cutting cost in comparison to piloted airplanes [9].

Combining mathematics and the stages of conventional photogrammetry [10,11] with computer vision methods, the structure from motion (SfM) technique [12] arose. SfM adjusts all bundles of all photos in such a way that the root mean square error (RMSE) in the projection of terrain points on the corresponding photos are minimized by the least squares method [13,14,15]. This optimization problem is known as bundle adjustment [16,17]. This has been developed based on two important publications: the book *Multiple View Geometry in Computer Vision* [18] and the scale invariant feature transform (SIFT) algorithm [19]. The former addresses the relative orientation problem from the projective geometry field, using the outlier concept (incorrectly paired points) in order to eliminate those points through random sample consensus (RANSAC) [20,21,22]. A large number of rows (homologous points) of matrix calculations is accelerated by sparse matrix techniques [23]. The SIFT algorithm, another important milestone, automatically identifies homologous points in the overlapping areas of photographs that have been covered (common areas of each photograph), with a success rate unexceeded since then. Lowe patented the SIFT algorithm and other researchers have studied different algorithms and found similar results [24]. 

Roads, being part of civil infrastructure, require conservation and maintenance that save time and thus cost. The first step in this process, determining the current state of the pavement, is possible by analyzing the point cloud (PC) from photogrammetry–RPAS. Thus, it is useful to analyze the influence of variables involved in the accuracy of PC generation over the asphalt shape and identify the most influential one. The key variables for PC accuracy, and consequently the ones studied here, are focal length, ground sample distance (GSD), overlap, and modulation transfer function (MTF). The choice of these variables was based on previous research: Rosnell and Honkavaara [25] investigated methods for PC generation combining different overlaps and using different cameras; Hernandez-Lopez et al. [26] analyzed geometrical features based on GSD and overlap that should be taken into account to make a photogrammetric flight plan; Mesas-Carrascosa et al. [27] concluded that the GSD must be taken into account to make a photogrammetric flight plan; Westboy et al. [28] recommended taking into account the overlap to generate PC photogrammetry; Tahar [29] analyzed different photogrammetric methods to obtain PCs combining different focal lengths; Näsi et al. [30] inspected anomalous reflectance characteristics of trees with hyperspectral cameras combining different GSDs; and Eltner and Schneider [31] studied the quality of DEMs generated from different cameras and different focal lengths. In addition, from our experience, we noted that the sharpest images produced better PC quality. So, sharpness should be included in our study. From this, we decided that a variable that could measure this physical phenomenon is the modulation transfer function 50 (MTF50).

It is necessary to know the camera’s internal features to develop PCs from camera images using photogrammetric techniques [32]. These features can be retrieved through the camera calibration process. The internal features involve the coordinates of the image projection center (xp, yp), the focal length (f), the radial distortion coefficients (k1, k2, k3), and the decentering lens distortion (p1, p2) [33]. The camera calibration process has been carefully analyzed in recent years and many techniques have been developed [34,35].

Variables are defined as follows: (a) Focal length is the distance in millimeters from the back nodal point to the point where light rays entering the lens converge to form a sharp image on the sensor. Depending on the focal length, any lens induces a spherical distortion in the resulting photo [36]. (b) GSD is the distance between pixel centers from a digital photo measured in millimeters on the ground. (c) MTF is determined from modulation of the Fourier transform applied over any time or any space domain, for example, to the speech field [37], to the study of response physics systems [38,39], or to photography, in order to measure lens sharpness [40,41]. In recent years, MTF has been analyzed to improve measures obtained from photogrammetry techniques [42,43,44,45,46]. (d) Overlap is the percentage of one photo covering another over the same ground terrain.

The influence of several variables over other variables or behavior can be analyzed by multiple regression to develop systems models, multivariate analysis of variance (MANOVA), principal component analysis (PCA), etc. Garcia-Balboa et al. [47] proved that artificial neural network (ANN) is the most suitable method for training complex datasets; this is the reason we selected this method. For the present purpose, ANN fulfills the dual purpose of analyzing and estimating accuracy for this system. Also, this ANN may serve as the beginning of an open global ANN to estimate the accuracy of a PC through a large data server on the web and the addition of patterns (input variables, output accuracy) from different cameras and lenses that train the ANN and introduce feedback.

When a complete 3D reconstruction of a highway network is needed, the storage capacity should be prepared for managing large amounts of data. Software currently exists to deal with this kind of large data problem, e.g., Autodesk, Bentley, Agisoft Photoscan, and Pix4D.

## 2. Methodology

**Figure 1:**
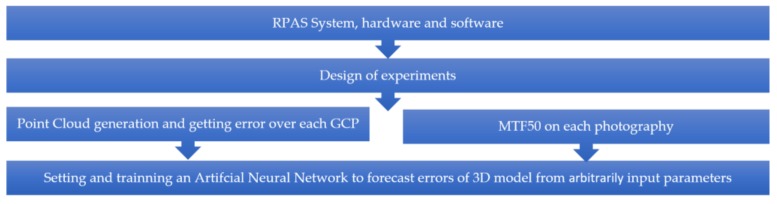
Research workflow. RPAS, remotely piloted aircraft systems; GCP, ground control point; MTF50, modulation transfer function 50.

The methodology followed throughout this research is presented in Figure 1.

### 2.1. RPAS System, Hardware, and Software

**Figure 2:**
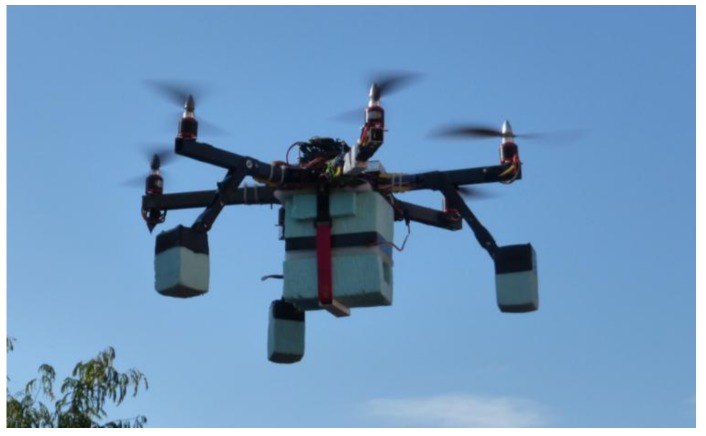
RPAS.

The RPAS platform used in this study is a multirotor hexacopter (Figure 2) developed by the authors. This system is designed to take zenith photographs. The RPAS payload is a small-format nonmetric digital camera Lumix tz35 (4608 × 3456 pixels, Live MOS 1/2.33″, Leica DC Vario-Elmar lens), serial number FA3KA001057, manufactured in Tokio (Japan). The RPAS payload is stabilized by a gimbal system to improve the accuracy of the camera’s exterior orientation parameters [48]. The hexacopter has onboard a navigation system based on: (i) a u-blox LEA6 global positioning system (GPS) receiver, (ii) an inertial measurement unit (IMU) system with a 3-axis gyro and accelerometer, (iii) an MS5611 barometric pressure sensor, and (iv) a microcontroller based on Arduino (ArduPilotMega 2.5), manufactured in Torino (Italy). With these avionics, the hexacopter can fly autonomously through a predefined set of waypoints. To set the fly-by waypoints, the authors developed a script to generate a track under the conditions required in each flight plan. The track format is compatible with the ground station software used to operate the RPAS, Mission Planner 1.2.85. The script takes flight variables as inputs (start and end flight points, overlap, and GSD desired) and track format as output. The script calculates the flight height and the points where the RPAS camera has to take photos over the track flight.

### 2.2. Experiments Design

The variables studied here are focal length, ground sample distance, modulation transfer function, and longitudinal overlap. The first 3 variables are related to the photo, and the PC is developed from a photo set so that the way in which each photo is taken will influence the PC.

The last variable, overlap, is related to the common pixels on every 2 photos so it can influence the resulting PC.

**Figure 3:**
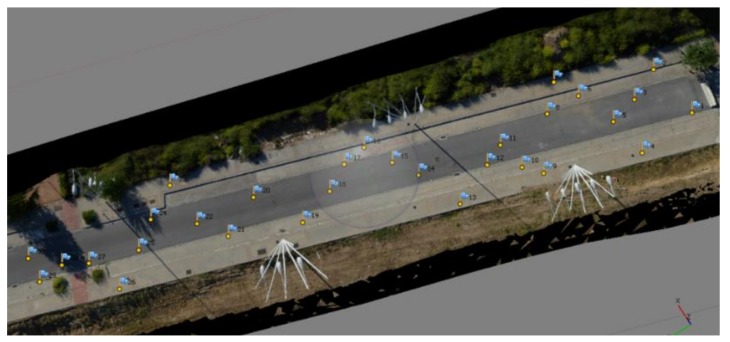
Flight test zone.

This study was made on a roadway cut for traffic into the city of Granada (S Spain). The test-field road measured about 100 × 10 m^2^. Specifically, the area was over 37.186539° N, −3.627872° geographical coordinates (latitude, longitude). On the test-field road, we randomly placed 30 ground control points (GCPs), maintaining the same initial random method throughout several light tests (Figure 3) to determine ground truth. The coordinates of each GCP were established by total station measures, which enabled positioning accuracy to 5 mm. Thus, each GCP represents a measure of known coordinates.

We performed 15 kinds of flight mission (Table 1). Each type of flight mission was repeated 4 times to ensure statistically valid results. Thus, 60 flight mission samples were gathered. The variables of each flight mission (focal length, GSD, and overlap) were imposed as flight and camera constraints.

The design of each flight required writing the mission in the RPAS. Before the mission was upgraded to the RPAS, it was necessary to apply the script developed in order to: (a) direct the flight along the planned trajectory and (b) activate the trigger in the specific place of the zenith photograph desired.

Also, the flights were executed with common parameters (the same in all flights). Also, as the photographs were taken in motion, the highest speed shutter possible was required (1/2000 s) and ISO 100 was used to ensure minimum noise. These are the maximum values offered by the Lumix tz35. Because of the shutter speed and ISO required, the aperture had to be opened as much as possible, i.e., F3.4, to admit maximum light.

### 2.3. Development of PC and 3D-RMSE Computation

**Figure 4:**
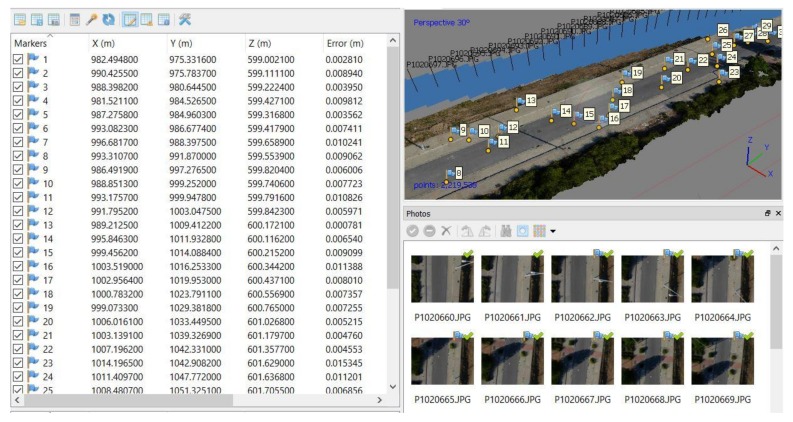
Development of the point cloud (PC). The first column shows the GCP number, the following three show the real GCP (ground truth), and the last one shows the square error between the PC generated by photogrammetry with respect to the ground truth.

After making 60 packs of photographs, each PC was developed to get the RMSE of each GCP in the PC. The software used to determine the PC and RMSE was Agisoft PhotoScan 1.1. The self-internal orientation or self-calibration and relative orientation were performed by the computer program within the same process, generating a PC in relative coordinates. Then, the data from the absolute coordinates (ground truth) of each of the 30 GCPs was imported to develop the PC in absolute coordinates, with their respective error calculated by Agisoft PhotoScan based on the calculation of the 3D-RMSE (Figure 4): $3D\_RMSE=\sqrt{RMSE_{x}^{2}+RMSE_{y}^{2}+RMSE_{z}^{2}}$.

### 2.4. MTF50

**Figure 5:**
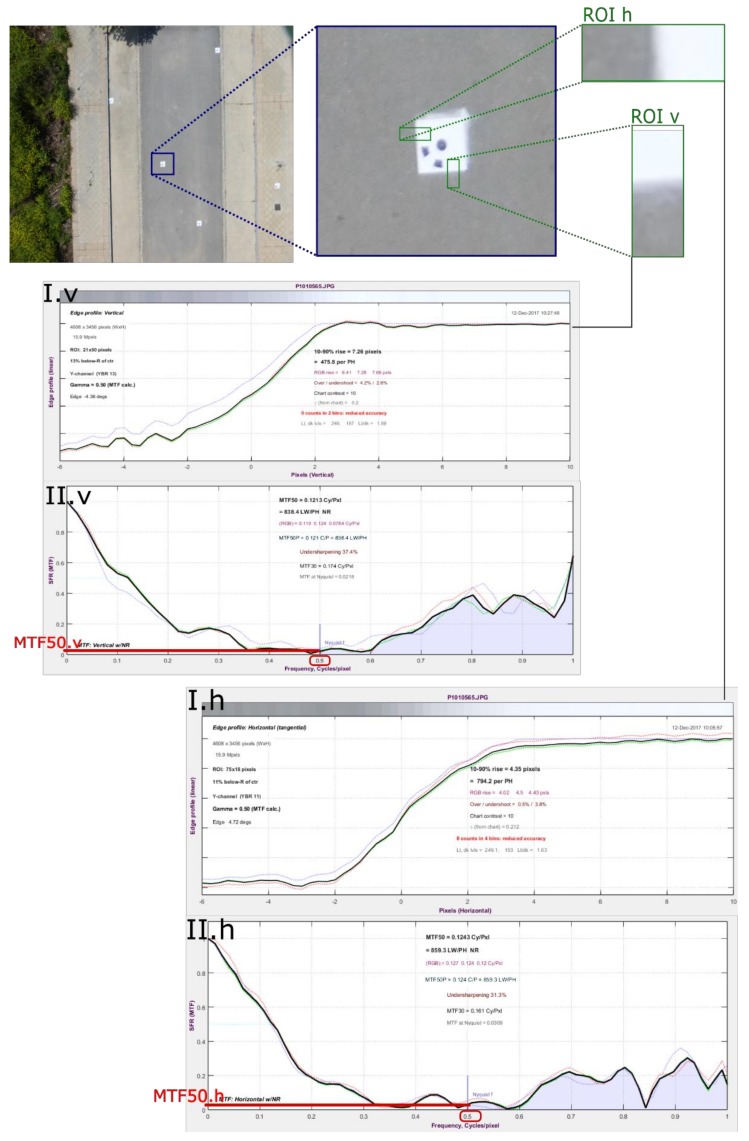
Workflow to determine the MTF50 of each photo(unamgraph on vertical and horizontal axes.

In this study, we needed to include certain variables related to the sharpness and contrast image. This is because the images are taken in motion and sharpness and contrast can change and contaminate neighboring pixels, causing disarray in the homologous pixel identification algorithm. Thus, we analyzed the MTF50. MTF is the spatial frequency response (SFR) of an imaging system or a component; it is the contrast at a given spatial frequency relative to low frequencies. MTF can be measured in Hertz or in cycles (or lines) per millimeter (or pixel). To get the MTF50, we followed the flowchart in Figure 5. First, we defined 2 regions of interest (ROIs) for each target (the space composed by asphalt and the white portion of the targets). Each photo has at least 1 target, so each one has at least 2 ROIs from the target. Then, each ROI was transformed from red–green–blue (RGB) to grayscale. Afterward, we made the grayscale average curve in the horizontal space domain of ROI.H to get I.h and the average curve in the vertical space domain of ROI.V to get I.v (see Figure 5). Next, we established the modulation transfer function from I.h and I.v to get II.h and II.v. Then, we registered the MTF value corresponding to the frequency of 0.5, which is the MTF50. Finally, the average value between MTF50.h and MTF50.v was calculated, and this was the MTF50 value on each photograph of each flight.

### 2.5. Setting and Training a Neural Network to Forecast 3D-RMSE of 3D Model from Arbitrary Input Parameters

**Figure 6:**
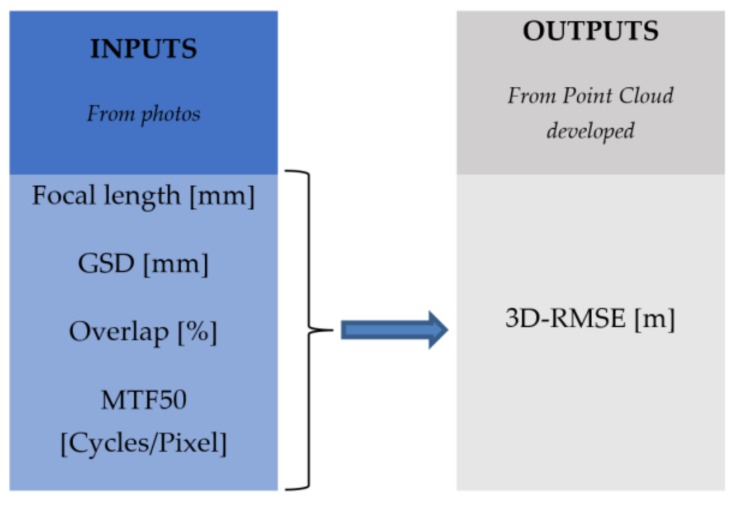
Artificial neural network inputs–output.

The basic aim of this project was to forecast the 3D-RMSE of PCs developed by the photogrammetric flight mission under certain controlled conditions (Figure 6). The variables in the project methodology were focal length, GSD, longitudinal overlap, and MTF50. For this, an artificial neural network back propagation was designed, set, trained, validated, and tested by MATLAB r2012b.

The variables monitored were the input neurons of the ANN, while the square 3D-RMSE was the output neurons of the ANN. The optimal number of hidden layers was not known beforehand and was determined by trial and 3D-RMSE. As this was not a complex problem, the ANN had a better fit when it was set with a smaller number of hidden layers [49]. The problem presented here with a single neuron in the output layer does not seem to be very complex. Therefore, it began with testing a single hidden layer, and when the convergence was deemed satisfactory, this design was validated.

In summary, the data were as follows:

- There were 60 photographic packs under different conditions controlling the variables under study. Each photographic pack generated a PC, so that 60 PCs were generated. Each PC had 30 GCPs. The 3D-RMSE of each GCP in each PC was determined. Then, we calculated the 3D-RMSE average of each 6 GCPs (by homogeneous zones), so we got 5 3D-RMSE samples of each flight (or each PC).
- The MTF50 in each photo on the x-axis and the y-axis was measured in order to use their average in the ANN.
- Based on the above, 60 (PC) × 5 (3D-RMSE samples GCP/PC) = 300 samples. Therefore, 300 samples were generated to train, validate, and test the ANN. Chosen randomly, 210 training samples, 45 validating samples, and 45 testing samples were used to set the ANN.

**Figure 7:**
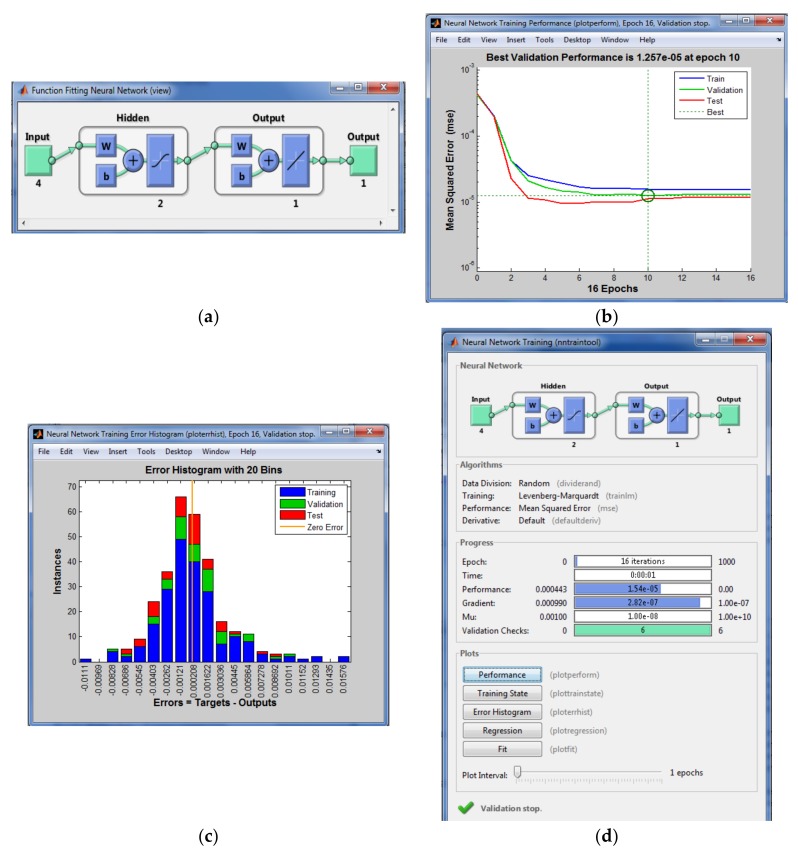
(**a**) Design of the ANN scheme; (**b**) mean square error (MSE); (**c**) error histogram shows the errors of each phase of ANN setting (training, validation, and testing); (**d**) training parameters of the ANN.

Several configurations were designed to train the ANN, and the configuration that worked best is shown in Figure 7a: 1 hidden layer with 2 neurons in this layer. The convergence was achieved with 10 epochs, converging with a mean square error (MSE) of 1.257·10^−5^ (Figure 7b). The errors in each epoch were near zero and the histogram that they comprise has a Gaussian shape around zero (Figure 7c). More information concerning the training process of the ANN is provided in Figure 7d.

## 3. Analysis and Discussion of the Results

**Figure 8:**
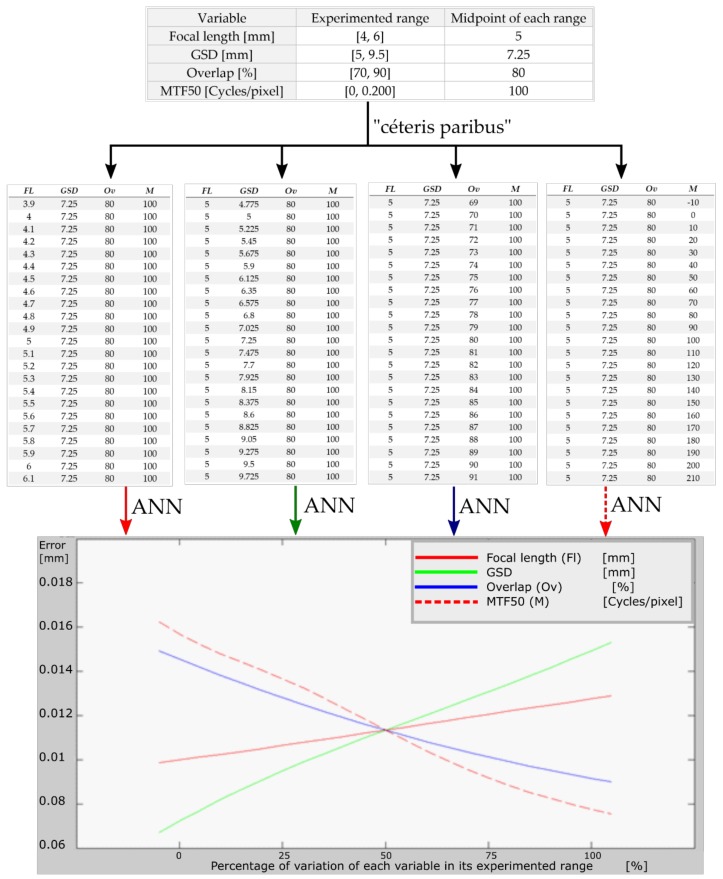
Influence of each variable on model accuracy.

Once the neural network was established, we proceeded to analyze it. The mean square error convergence value was 1.257·10^−5^ m. This value is less than a millimeter, indicating that when ordering values to the neural network, the response output will have an error of ±1.257·10^−5^ m. In addition, using the trained neural network, we determined which of the variables was the most influential on the output accuracy. To do so, we designed a *ceteris paribus* input table in which we calculated the midpoint value of the extreme values of the experienced range of each of the four variables. Then, we fixed the midpoint of each variable and changed 5% of the other variables. This was done for each of the four variables. These inputs we reused to compute the error by the neural network and obtained the graphics shown in Figure 8. Each line represents a variable of the four variables considered in this paper. The x-axis runs throughout the whole range in which each variable was studied, with 0, 50, and 100 being the beginning, midpoint, and end of each variable range, respectively. The y-axis gives the 3D-RMSE by computing the inputs in the neural network.

The growth of the curves in Figure 8 provides us with the following information: (a) when the MTF50 increases, the PC 3D-RMSE decreases; (b) when the GSD increases, so does the PC 3D-RMSE; (c) when overlapping increases, the PC3D-RMSE decreases; and (d) when focal length increases, so does the PC 3D-RMSE (as expected). Thus, we might deduce that any improvement in the average sharpness of the photographs developed from the PC will reduce the 3D-RMSE, meaning that the accuracy of the PC would be better. This is because the software can better identify each pixel and thus can provide a model closer to reality. The same is true of the GSD or ground pixel size—that is, as might be expected, the larger the pixel, the less ground information will be obtained and the coarser the data, therefore the software will have poorer geometrical quality information to develop the PC. Also, the more longitudinal overlap, the better the PC will be, because the software has more homologous points between two consecutive images, and thus more accurate PC results.

After analyzing the graph of Figure 8, we calculated the best least square to fit a line through the output neural network 3D-RMSE of each variable in order to determine the variable that most influenced the 3D-RMSE of the PC developing process. The slopes of the resulting line were: (a) MTF50: −4.1·10^−4^; (b) GSD: 3.7·10^−4^; (c) overlapping: −2.6·10^−4^; and (d) focal length: 1.2·10^−4^. Taking into account the slope change, we propose that the most changing variable over its range and therefore the variable that most influences the accuracy of a PC on asphalt is the MTF50 of each photograph, followed by GSD, overlapping, and focal length.

Additionally, we take into account the importance of the MTF50 linked to each image, instead of the traditional analysis of it as a measure belonging to the camera system (lens). The MTF50 can provide a measure related to blurring, moving, and sharpness of an image because this value is related to light and dark image changes. This idea is far from the traditional treatment of MTF50 values.

## 4. Conclusions

The method used in this study determined accuracy when using photogrammetry to compute the point cloud (PC) over asphalt, and it is similar to total station accuracy. In particular, the average accuracy found was about ±1 cm. Therefore, this method is suitable for photogrammetric mapping in asphalt areas to support decision-making about the needs for renovation, conservation, and maintenance of road surfaces.

It has been demonstrated that the most influential variable in the accuracy of the PC is the MTF50, representing a measure of sharpness. In other words, establishing an accurate PC requires the use of cameras with suitable MTF50 values. It is also predicted that a key feature to improve MTF50 is shutter speed, because the images are taken with the RPAS in motion. Thus, the speed should be greater than 1/2000 s. We recommend further research on how to improve the MTF50 of a photographic shot with a moving camera or a vibrating subject.

The next variable to consider is the GSD. With other variables fixed, flight at lower altitudes provides greater accuracy in the PC. This presents a drawback: the area photographed will be smaller. Therefore, we recommend the use of a nonmetric camera with the image sensor as large as possible to guarantee higher flights without loss of GSD accuracy.

According to the classical ANN approach, the comparison between the 3D-RMSE estimated by the fitted ANN and the test subset suggests that ANN is a suitable tool for estimating the 3D-RMSE when fed by the following variables: MTF50, GSD, overlap, and focal length. We developed a tool that can advise as to the values those variables should take in order to ensure predefined accuracy. For future research, the tool has the advantage of being able to add more patterns to the neural network, and by training, validating, and testing, it can also expand the range of variables.

Mapping with RPAS has proven to be easier and more versatile than conventional topography and photogrammetric flight data collection with an airplane. It is especially remarkable when the mapping methods are used to sweep the length of intermediate areas between standard topography (small areas) and conventional photogrammetric flights (large areas).

## Figures and Tables

**Table 1:** Set of flight missions. GSD, ground sample distance.

Flight Mission	Focal Length (mm)	GSD (mm)	Overlap (%)
*1*	4	**7**	**90**
*2*	4	7	80
*3*	4	7	70
*4*	6	5	90
*5*	6	5	80
*6*	6	5	70
*7*	4	5	90
*8*	4	5	80
*9*	4	5	70
*10*	6	7	90
*11*	6	7	80
*12*	6	7	70
*13*	4	9.5	90
*14*	4	9.5	80
*15*	4	9.5	70
